# Aquaporin-9 Contributes to the Maturation Process and Inflammatory Cytokine Secretion of Murine Dendritic Cells

**DOI:** 10.3389/fimmu.2018.02355

**Published:** 2018-10-16

**Authors:** Stefania De Santis, Grazia Serino, Maria R. Fiorentino, Vanessa Galleggiante, Patrizia Gena, Giulio Verna, Marina Liso, Monica Massaro, Jinggang Lan, Jacopo Troisi, Ilaria Cataldo, Alessia Bertamino, Aldo Pinto, Pietro Campiglia, Angelo Santino, Gianluigi Giannelli, Alessio Fasano, Giuseppe Calamita, Marcello Chieppa

**Affiliations:** ^1^National Institute of Gastroenterology “S. de Bellis”, Research Hospital, Castellana Grotte, Italy; ^2^Department of Pharmacy, University of Salerno, Fisciano, Italy; ^3^Pineta Grande Hospital, Castelvolturno, Italy; ^4^Harvard Medical School Division of Pediatric Gastroenterology and Nutrition and Mucosal Immunology and Biology Research Center, Massachusetts General Hospital for Children, Boston, MA, United States; ^5^Department of Biosciences, Biotechnologies and Biopharmaceutics, University of Bari “Aldo Moro”, Bari, Italy; ^6^Department of Medicine and Surgery and Dentistry, “Scuola Medica Salernitana”, University of Salerno, Salerno, Italy; ^7^Theoreo srl-Spin-off Company of the University of Salerno, Salerno, Italy; ^8^European Biomedical Research Institute of Salerno, Salerno, Italy; ^9^Institute of Sciences of Food Production C.N.R., Unit of Lecce, Lecce, Italy

**Keywords:** AQP9, dendritic cells, inflammation, il-12, glycerol

## Abstract

Dendritic cells (DCs) are the most potent antigen-presenting cells able to trigger the adaptive immune response to specific antigens. When non-self-antigens are captured, DCs switch from an “immature” to a “mature” state to fulfill their function. Among the several surface proteins involved in DCs maturation, the role of aquaporins (AQPs) is still poorly understood. Here we investigated the expression profile of *Aqps* in murine bone marrow derived dendritic cells (BMDCs). Among the Aqps analyzed, *Aqp9* was the most expressed by DCs. Its expression level was significantly upregulated 6 h following LPS exposure. Chemical inhibition of Aqp9 led to a decreased inflammatory cytokines secretion. BMDCs from AQP9-KO mice release lower amount of inflammatory cytokines and chemokines and increased release of IL-10. Despite the reduced release of inflammatory cytokines, Aqp9-KO mice were not protected from DSS induced colitis. All together, our data indicate that AQP9 blockade can be an efficient strategy to reduce DCs inflammatory response but it is not sufficient to protect from acute inflammatory insults such as DSS induced colitis.

## Introduction

Dendritic cells (DCs) are the most potent antigen-presenting cells, specialized in recognizing pathogens, presenting antigens and triggering the acquired immune response (1). DCs are a heterogeneous class of cells originating from hematopoietic progenitors and differentiate in various anatomic locations to fulfill different functions (2). Immature DCs (iDCs) exhibit a high endocytic activity, critical for sampling the environment in search of potential threats to the host. Once activated, DCs undergo a maturation process, characterized by marked changes in gene and protein expression, cytokines and chemokines secretion and cell biology (3). Mature DC (mDCs) play a central role in the adaptive immune response, by presenting antigens and driving the activation and the polarization of antigen-specific T cells via cytokines secretion (4). DCs maturation involves profound metabolic changes, involving in particular energy production, required to support chemotaxis and biosynthesis of soluble factors (5). Among the numerous surface proteins expressed by DCs, acquaporins (AQPs) have been only marginally studied (6, 7). Protein-dependent water transport was first described in 1987 (8) when Aquaporin1 (AQP1) was purified from red blood cells. Since then, a 13-member family of AQPs (9) has been identified. AQPs are membrane proteins that act as channels for water and other small solutes (including glycerol, lactate, and purines) transport across the membrane. (8, 10, 11). Mammalian AQPs are currently divided into two different subgroups: aquaporins, that include AQP0, 1, 2, 4, 5, 6, and 8 and aquaglyceroporins, that include AQP3, 7, 9–12 (12). This second group facilitate the transport of water and small neutral solutes across cell membranes. AQPs expression on immune cells was previously associated only with antigen uptake and endocytosis (13), but recent studies have demonstrated that AQP expression is also crucial for immune cell migration (14, 15), cytokines release (16) survival, and homeostasis (17).

We have recently described the anti-inflammatory effects of murine bone marrow-derived dendritic cells (mBMDCs) after exposure to quercetin (18–20). Polyphenols treatment was also able to induce an alternative DCs surface protein expression (21). Among the several genes modulated by quercetin, we identified *Aqp*9 as the only member of the AQPs family expressed by BMDCs following LPS administration.

Here we explore AQPs expression in murine DCs, cultured *in vitro* or purified from different lymphoid compartments. We show that *Aqp*9 is frequently the most expressed *Aqp* in DCs, and its expression is upregulated following LPS exposure. *Aqp9* chemical inhibition (22) reduces inflammatory cytokines secretion. Therefore, we compared WT and AQP9-KO BMDCs maturational profiles, demonstrating that AQP9 is required for efficient DCs maturation and to trigger the inflammatory response.

## Results

### Aqp9 is the only member of the AQP family to be modulated by LPS in murine BMDCs

**Figure 1:**
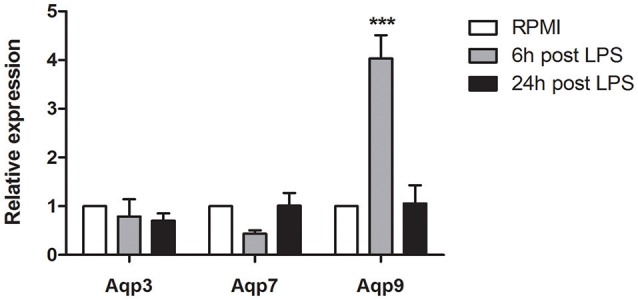
Dendritic cells *Aqps* expression. The expression of *Aqp*3, *Aqp*7 and *Aqp*9 was assessed at 6 and 24 h after the stimulation with LPS (1 μg/mL). Results are expressed as fold change compared to immature BMDCs. Bars represent the mean ± SEM of 3 independent experiments. ***P < 0.001.

In our previous study, we addressed the gene expression profile of polyphenols pre-treated mBMDCs after LPS administration by performing a microarray analysis (18–21). Supplementary Table 1 shows that administration of the Quercetin and Piperin Reconstituted Oil Bodies (ROBs-QP) significantly increased the AVG signal for *Aqp*9 in BMDCs, 6 h after LPS administration. Of note, the AVG signal of all other Aqps (*Aqp*1-12) was much lower and often below the detection limit (Supplementary Table 1). Based upon our microarray data, we evaluated the expression of several members of the AQP family (*Aqp*1, *Aqp*3, *Aqp*5, *Aqp*7, *Aqp*9) by qPCR in immature and LPS-treated DCs, at 6 and 24 h after LPS stimulation. Figure 1 shows a significant increase of *Aqp*9 expression 6 h after LPS administration while no significant changes were detected for both *Aqp*3 and *Aqp*7. *Aqp9* expression returned to baseline level 24 h after LPS exposure. Surprisingly, the expression of *Aqp*1 and *Aqp*5 was consistently below detection. As this observation is in contrast with previously reported data we validated the efficiency of the primers used for the qPCR. The mRNA obtained from the lung of 3 WT mice was tested for *Aqp*1 and *Aqp*5 expression. The results obtained confirmed the efficiency of the selected primers (Supplementary Figure 1).

### *Aqps* expression in murine DCs from different tissues

**Figure 2:**
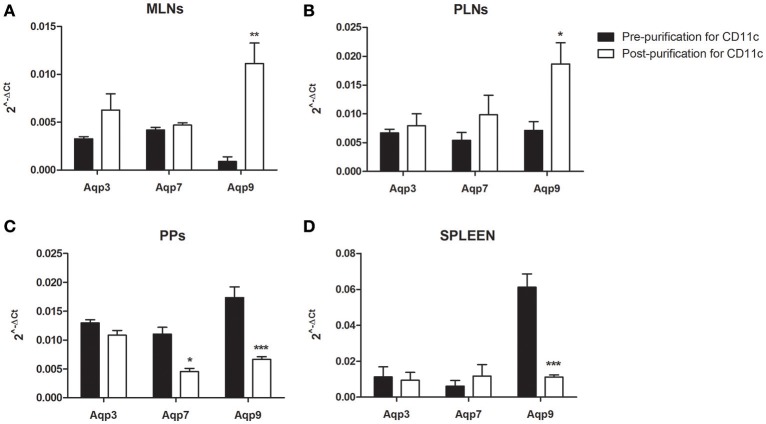
Expression analysis of *Aqps* in different lymph nodes. The expression of *Aqp*3, *Aqp*7, and *Aqp*9 was assessed in Mesenteric Lymph Nodes (MLNs) **(A)**, Peripheral Lymph Nodes (PLNs) **(B)**, Peyer's Patches (PPs) **(C)**, and spleen **(D)** of WT mice. Bars represent the mean ± SEM of 3 independent experiments. *P < 0.05, **P < 0.01, ***P < 0.001.

Recent studies have addressed *Aqps* expression in distinct immune cells, suggesting that DCs from different anatomical compartments have a different *Aqps* repertoire (13–17, 23). We investigated the expression of *Aqp*1, *Aqp*3, *Aqp*5, *Aqp*7, *Aqp*9 by qPCR in different lymphoid compartments including mesenteric and peripheral lymph nodes (MLNs and PLNs, respectively), Peyer's Patches (PPs) and spleen. Among AQPs, *Aqp*9 appeared to be the most expressed in spleen and PPs, while *Aqp*3 and *Aqp*7 were more strongly expressed in the MLNs (Figures 2A,C,D, black bars). The same analysis was performed focusing on purified DCs from the above tissues using CD11c^+^ cells magnetic enrichment. CD11c^+^ cells from the MLNs and PLNs express high *Aqp*9 expression level as compared to *Aqp*3 and *Aqp*7 (Figures 2A,B, white bars). The opposite trend was observed in the CD11c^+^ cells from PPs and spleen; in fact *Aqp*9 expression resulted lower than in the whole tissue following CD11c^+^ cells enrichment (Figures 2C,D). Of note, the expression level of *Aqp1* and *Aqp5* was consistently below detection both in all samples analyzed.

### Aqp9 chemical inhibition reduces DCs ability to release inflammatory cytokines

**Figure 3:**
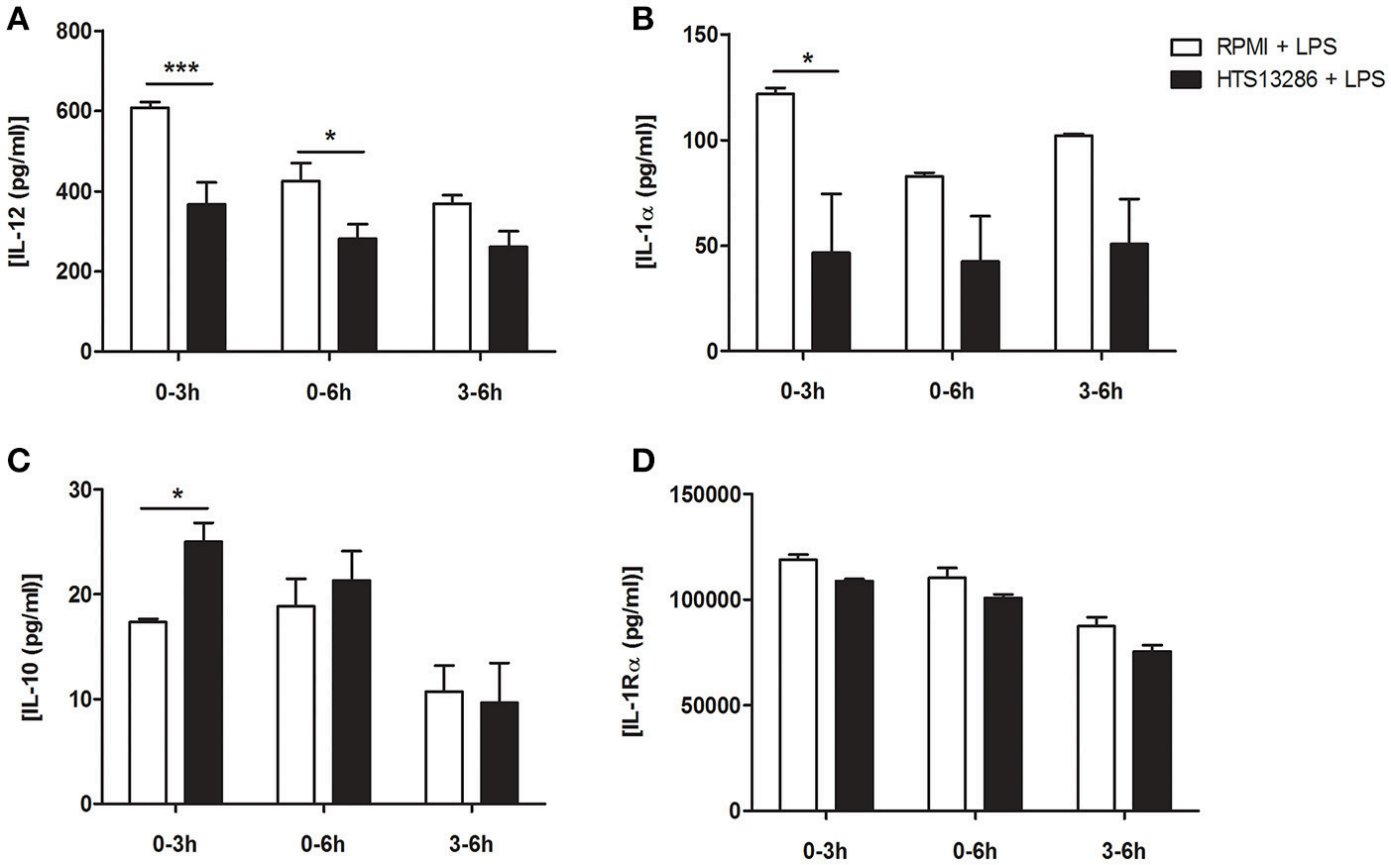
HTS13286 treatment reduces the inflammatory abilities of DCs. **(A–D)** Mature BMDCs were exposed to HTS13286 at the indicated times. Pro/anti-inflammatory cytokines secretion (IL-12 and IL-1α/IL-10 and IL-1Rα, respectively) was determined 24 h later by ELISA. *P < 0.05, **P < 0.01, ***P < 0.001.

To evaluate the biological significance of *Aqp*9 expression during DCs maturation, we investigated whether a chemical blockade of AQP9 with a specific inhibitor, HTS13286 (22), could affect DCs response to an inflammatory stimulus. We focused on the modulation of the inflammatory response. In a preliminary dose-response study, we identified 25 μg as the most efficient concentration for AQP9 inhibition in BMDCs, in line with what has been previously reported using macrophages (22). Furthermore, we observed a stronger reduction of inflammatory cytokines release after two consecutive administrations of the chemical inhibitor (Supplementary Figure 2). We then tested different time points for the administration of the inhibitor: 0 and 3 h, 0 and 6 h, 3 and 6 h after LPS stimulation. BMDCs supernatants were collected 24 h after LPS administration and analyzed by ELISA. Figures 3A–D indicates the decreased ability of LPS-stimulated DCs to release pro-inflammatory cytokines after AQP9 inhibition with the two stimulations at 0 and 3 h (IL-1α and IL-12, Figure 3A,B). IL-1Rα was not reduced by HTS13286 administration while IL-10 was slightly but significantly increased after the HTS13286 administration at the same time point (Figures 3C,D).

To investigate whether AQP9 inhibition by HTS13286 could perturb the cell numbers or surface expression of MHCII we performed FACS analysis. Cells analyzed by FACS revealed that the percentage of MHCII^+^ DCs was not altered after AQP9 blockade. A non-significant tendency to a reduction in MHCII expression was observed (Supplementary Figure 3).

### Unique maturational profile for Aqp9-KO DCs

**Figure 4:**
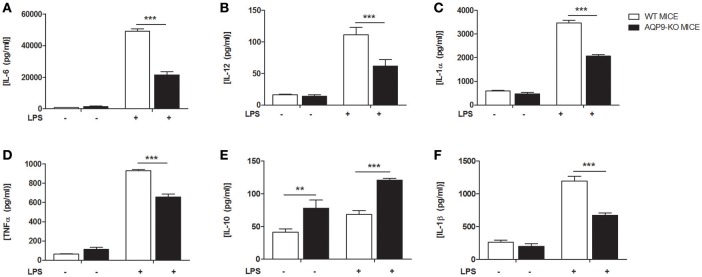
AQP9-KO DCs cytokine profile following LPS exposure. BMDCs from WT and Aqp9-KO mice were exposed to LPS for 24 h. **(A–F)** Cytokine **(**IL-6, IL-12, IL-1α, TNFα, IL-10, IL-1β) concentrations were determined in the supernatant by ELISA. Bars represent mean ± SEM of 3 independent experiments. **P < 0.01, ***P < 0.001.

**Figure 5:**
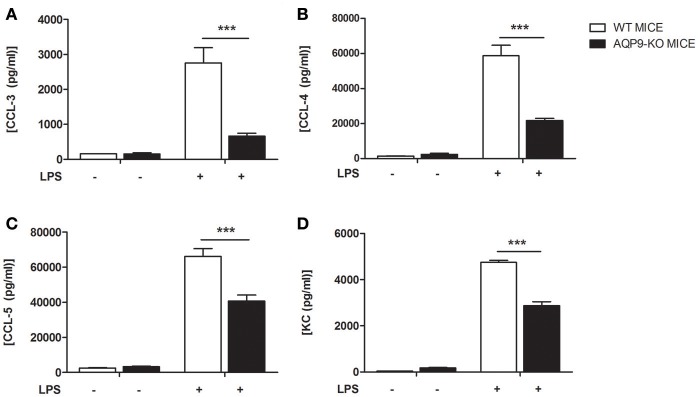
AQP9-KO DCs chemokine profile following LPS exposure. BMDCs from WT and Aqp9-KO mice were exposed to LPS for 24 h. **(A–D)** Chemokine (CCL-3, CCL-4, CCL-5, and KC) concentrations were determined in the supernatant by ELISA. Bars represent mean ± SEM of 3 independent experiments. ***P < 0.001.

**Figure 6:**
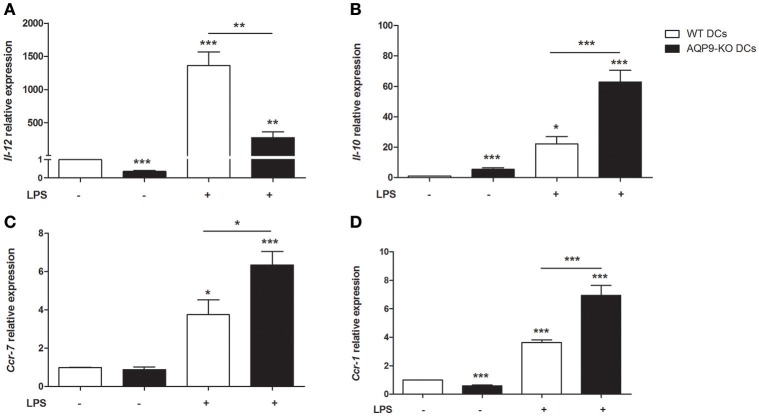
Expression analysis of Aqp9-KO DCs. The expression of *Il-12, Il-10, Ccr-7*, and *Ccr-1* was assessed in BMDCs of WT and Aqp9-KO mice **(A–D)**. Bars represent the mean ± SEM of 3 independent experiments. *P < 0.05, **P < 0.01, ***P < 0.001.

As HTS13286 was only partially able to block AQP9, we decided to use AQP9-KO bone marrow-derived DCs (23) and investigated the effects of the AQP9 knockout during LPS-induced DCs maturation. WT and AQP9-KO DCs were cultured *in vitro* and exposed to LPS to evaluate the release of cytokines and chemokines and the expression of selected genes involved in DCs maturation. AQP9-KO DCs secrete lower concentrations of IL-6, IL-12, IL-1α, TNFα, IL-1β, (Figures 4A–D,F), CCL-3,−4,−5, and KC (Figure 5) following LPS administration. Surprisingly, the anti-inflammatory cytokine IL-10 was increased in the supernatant of AQP9-KO (Figure 4E). The expression levels of *Il-12* and *Il-10* confirmed what observed in the supernatant of AQP9-KO DCs (Figures 6A,B). The same samples were used to address the expression of genes involved in DCs maturation. The up-regulation of *Cd-80, Cd-86*, and *Cd-274* was similar in WT and AQP9-KO DCs exposed to LPS (data not shown). *Ccr-7* expression was increased 6 h after LPS exposure, while *Ccr-1* expression was increased and not reduced in AQP9-KO DCs, suggesting an alternative maturational pathway in these DCs (Figures 6C,D).

### Absence of Aqp9 does not protect from DSS induced colitis

**Figure 7:**
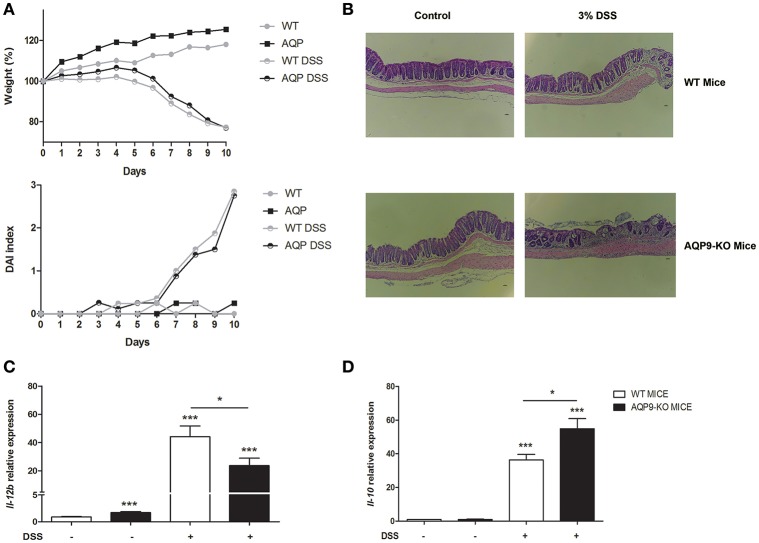
Experimental colitis in Aqp9-KO mice. Acute inflammation was induced by 3% of DSS for 7 days, followed by 4 days of recovery. Weight was recorded every day **(A)**, at day 11 colon morphology was analyzed **(B)**. The expression of *Il-12* **(C)** and *Il-10* **(D)** was assessed in the colon of WT and Aqp9-KO mice. *P < 0.05, ***P < 0.001.

With the intent to evaluate the importance of Aqp9 expression in a model of acute inflammation, we used 3% DSS to induce colitis in AQP9-KO and WT mice. Figure 7A shows similar weight loss tendency induced by DSS in AQP9-KO and WT mice and no morphological differences were detected by histological analysis (Figure 7B).

Molecular analysis of inflammatory gene expression on WT and AQP9-KO colon samples confirmed the *in vitro* data revealing a significantly reduction of *Il-12* and, on the opposite, *Il-10* upregulation (Figures 7C,D).

## Discussion

A characteristic feature of the immune response is the plasticity of the immune cells. When required, immune cells migrate, divide, change their surface proteins, release soluble factors, switch their metabolism, die, or survive (14, 24–31). Among immune cells, dendritic cells play a pivotal role due to their antigen-presenting cell function (32). DCs can promote an inflammatory adaptive response to bacterial threats or sustain homeostasis and tolerance in mucosal tissues (33). As DCs migrate and reside in very different organs, it is not surprising that they acquire organ-specific markers (34, 35). Numerous surface markers have been reported for different DCs subsets, including AQPs, that were first described by De Baey and Lanzavecchia (6). The expression of AQPs was at that time associated with micropinocytosis, endocytosis, and cellular swelling (6, 16, 36), but recently, it has emerged that AQPs, particularly aquaglyceroporins, are needed during inflammation to sustain the metabolic needs of the immune response (17).

For dendritic cells, the switch from immature to mature state involves profound changes in the cellular metabolic pathways, turning from oxidative phosphorylation to aerobic glycolysis (5). Literature on the involvement of some AQPs in these phenomena led us to focus our attention on AQP family (37). In this study, we report the *Aqp*-expression repertoire of murine DCs cultured *in vitro* from wild type mice bone marrow or purified from different lymphoid tissues. Unlike previous studies, *Aqp*9 was consistently the most strongly expressed Aqp found in BMDCs cultured *in vitro* after LPS stimulation. Our data are in line with what reported by de Baey et al. using human DCs cultured from circulating monocytes (6), but in contrast with Moniaga et al. that report very low Aqp9 expression in immature bone marrow derived DCs (15). Furthermore, we confirmed DCs expression of Aqp3 and Aqp7 reported by Moon et al. (7), but failed to detect Aqp5 expression that was previously reported by Wang et al. (13). We confirmed the efficiency of the primers used for our analysis using lung extracted mRNA as this tissue has been reported to express high levels of Aqp1 and Aqp5 by the Entrez database (https://www.ncbi.nlm.nih.gov/gene/11826; https://www.ncbi.nlm.nih.gov/gene/11830). It is tempting to speculate that different culture protocols and reagents may be responsible for the discrepancies in Aqp5 expression, but further experiments are required to sustain this hypothesis. In line with this, *Aqp9* expression levels also resulted higher in CD11c magnetically-enriched cells from mesenteric and peripheral lymph nodes suggesting that CD11c^−^ cells from these lymph nodes express low levels of *Aqp*9. On the contrary, in the PPs and spleen the whole tissue expressed higher *Aqp9* levels than the CD11c^+^ enriched-population. FACS analysis of the CD11c enriched cells revealed that only a minor contamination from MHCII^−^Ly6G^+^ cells was present in samples obtained from the spleen (Supplementary Figure 4A). We then tested *Aqp9* expression level in DCs progenitors. Supplementary Figure 5 shows a tendency to increase *Aqp9* expression level during DCs maturation (from day 3 to day 7). Day 3 samples containing 37.5% of MHCII^−^Ly6G^+^ express lower *Aqp9* level that day 7 cells containing only 9.4%, nonetheless, differences were non-significant to explain the *Aqp9* decrease in the spleen CD11c enriched cells. The majority of CD11c^+^ cells was CD11b^+^, while F4/80 was expressed by approximately 20% of the CD11c^+^ cells obtained from the spleen, 10% of the PLNs and by a minor population of the MLNs and PPs enriched cells. CD103^+^CD11b^−^ cells were present only in the PPs enriched cells (Supplementary Figure 4B). We interrogated the published dataset by Rivollier et al. (38) to compare Aqp9 expression in macrophages and dendritic cells sorted from the colon, but in the published dataset no significant differences were reported. As the percentage of MHCII^lo^ was higher both in spleen and PPs after the CD11c magnetic enrichment (~40%), it is tempting to speculate that a contamination of DCs precursors in spleen and PPs samples may be responsible for differences in *Aqp9* expression. It is also possible that different DCs subpopulation express different Aqps repertoire, and more data will be required to address this question. The variation of *Aqp9* transcript expression following DCs maturation suggests that this AQP may play an active role in the maturational cascade that follows LPS stimulation. AQP9 is an aquaglyceroporin membrane channel whose role in transporting water, glycerol, and other small solutes has already been reported (23). As demonstrated by Phyu et al, DCs maturation induced by LPS administration requires glycolytic activity to supports their metabolic needs. The inhibition of glycogenolysis, leads to glucose reduction and attenuates DCs maturation (39). Glycerol is a crucial substrate for glucose synthesis, thus, upon the metabolic pressure dictated by the inflammatory condition, the inhibition of Aqp9 may result in DCs intracellular glycerol decrease and, consequently, reduced inflammatory ability. Following HTS13286 administration, inflammatory cytokines secretion and, in part, MCHII expression were reduced by AQP9 chemical inhibition. Our results are consistent with what has previously been published (16, 40), although our data were obtained by blocking a single AQP with a specific chemical inhibitor, and the overall DCs morphology was not different between treated and untreated cells.

To further explore the axis between AQP9 expression and the DCs maturational profile, we studied the bone marrow in AQP9-KO mice. LPS administration in the absence of AQP9 results in a decreased release of inflammatory cytokines and chemokines including IL-1α, IL-1β, IL-6, IL-12, TNFα, CCL3-5, and KC. Furthermore, AQP9 knock-down results in a perturbed chemokine receptor switch following LPS exposure. Similar to WT DCs, AQP9-KO DCs upregulate *Ccr7* expression in response to LPS, but fail to reduce *Ccr1* expression, possibly acting as a functional decoy for inflammatory chemokines (41). The concomitant decrease of IL-12 and increase of IL-10, both at molecular and protein level, may be the reason for the defective chemokine receptor switch observed. Differently from WT DCs, AQP9-KO cells increase Aqp7, but not Aqp3, expression level 24 h following LPS administration (Supplementary Figure 6) likely as a tentative to compensate AQP9 absence. Finally, AQP9 knock-down was not able to protect from DSS induced weight loss, although we observed a significant increase in *Il-10* expression level and reduced expression of *Il-12* in the colon of AQP9KO mice. DSS induced colitis is the result of an exposure to luminal antigens, thus, our molecular data obtained from the colon of DSS exposed mice are in line with the *in vitro* results previously discussed. DSS weight loss is the result of an acute insult to the intestinal mucosa, thus likely different models should be used to better explore the therapeutic potential of AQP9 pharmacological inhibition.

Overall, our results suggest that AQP9 expression is among the steps needed for DCs maturation in response to an inflammatory stimulation even if it is not sufficient to protect from DSS induced colitis. Based on recent observations, future studies will need to reveal the potential of AQP9 in the context of chronic inflammatory syndromes and may help to design new drugs that can efficiently block AQP9 contributing to suppress the inflammatory cascade.

## Materials and methods

### Mice

WT and Aqp9 KO (B6.129S1-Aqp9tm1Nlsn/Mmjax) murine lines were purchased from Jackson Laboratories (stocks No: 000664 and 37111, respectively). Animal experiments were carried out in accordance with Directive 2010/63/UE, enforced by Italian D.L. 26/2014, and approved by the Committee on the Ethics of Animal Experiments of the Ministero della Salute-Direzione Generale Sanità Animale (768/2015-PR 27/07/2015), the official RBM veterinarian and by the animal care and use Committee of the University of Bari (OPBA di Ateneo) and the Italian Ministry of Health (authorization n. 996/2015-PR). The Institutional Animal Care of Harvard Medical School (protocol #2013N000013) approved the protocol for the treatment of Aqp9 KO and WT mice with DSS. Animals were sacrificed if in severe clinical conditions, in order to avoid undue suffering.

### Generation and culture of murine BMDCs

BMDCs were obtained from WT and AQP9KO mice. Briefly, single cell suspensions of BMDCs from the tibiae and femurs of 6- to 8-week-old male mice were flushed with 0.5 mM EDTA (Thermo Fisher Scientific, MA, USA), and depleted of red blood cells with ACK lysing buffer (Thermo Fisher Scientific, MA, USA). Cells were plated in a 10 ml dish (1 × 10^6^ cells/mL) in RPMI 1640 (Thermo Fisher Scientific, MA, USA) supplemented with 10% heat-inactivated fetal bovine serum (FBS, Thermo Fisher Scientific, MA, USA), 100 U/mL penicillin (Thermo Fisher Scientific, MA, USA), 100 mg/mL streptomycin (Thermo Fisher Scientific, MA, USA), 25 μg/mL rmGM-CSF (Miltenyi Biotec, Bergisch Gladbach, Germany), and 25 μg/mL rmIL-4 (Miltenyi Biotec, Bergisch Gladbach, Germany) at 37°C in a humidified 5% CO_2_ atmosphere. On day 5, cells were harvested, re-stimulated with new growth factors and plated at 10^6^ cells/mL on 24-well culture plates. On day 7 BMDCs were stimulated with 1 μg/mL of LPS (L6143, Sigma-Aldrich, St Louis, MO, USA) and supernatants were collected 24 h after LPS stimulation. For the treatment with AQP9 inhibitor, BMDCs were treated with 25 μm of HTS13286 (Maybridge, Cornwall, UK) (22) at day 7, immediately after LPS stimulation. A second stimulation with HTS13286 was carried out 3 h after LPS.

### RNA extraction and qPCR analysis

Total RNA was isolated from BMDCs treated with LPS and HTS13286 and from different lymph nodes: Mesenteric Lymph Nodes (MLNs), Peripheral Lymph Nodes (PLNs), Peyer's Patches (PPs) and spleen of WT mice. The RNA was extracted using TRIzol® (Thermo Fisher Scientific, MA, USA) according to the manufacturer's instructions. The High Capacity cDNA Reverse Transcription kit (Thermo Fisher Scientific, MA, USA) was used to reverse transcribe 500 ng of total RNA, using random primers for cDNA synthesis. Gene expression of *Aqp1, Aqp3, Aqp5, Aqp7, Aqp9, Il-12, Il-10, Ccr-1, Ccr-7, Cd-80, Cd-86, Cd-274*, and *Gapdh* was tested with PrimePCR®Assay murine primers (Biorad, CA, USA): *qMmuCID0020860, qMmuCED0046868, qMmuCID0006880, qMmuCID0025269, qMmuCID0022285, qMmuCID0022404, qMmuCED0044967, qMmuCID0006862, qMmuCID0007010, qMmuCID0026745, qMmuCID0006086, qMmuCID0011907, qMmuCED0027497*, respectively. Real-time analysis was performed on the CFX96 System (Biorad, CA, USA) and the ΔCt and ΔΔCt method was used to calculate the absolute and relative expression, respectively.

### Enzyme-linked immunosorbent assay (ELISA)

Cell culture supernatants were analyzed for IL-12p70, IL-10, IL-6, TNF α, IL-1α, IL-1β, IL-1Rα, CCL3-5, and KC release in triplicate, using an ELISA kit (R&D Systems, Minneapolis, MN, USA) following the manufacturer's instructions.

### Cytofluorimetric analysis

BMDCs treated with LPS and HTS13286 were detached from the plate with DPBS 1X (Gibco, NY, USA) + 0.5 mM EDTA (Thermo Fisher Scientific, MA, USA), washed with DPBS 1X + 0.5 %BSA (Sigma-Aldrich, St Louis, MO, USA) and stained with CD11c PE (Miltenyi Biotec, Bergisch Gladbach, Germany) and MHCII APC (Miltenyi Biotec, Bergisch Gladbach, Germany). Flow Cytometer acquisition was performed using NAVIOS (Beckman Coulter, CA, USA).

### DSS induced colitis

To induce colitis, 4–6 weeks old mice were given 3% (w/v) DSS (molecular weight 36,000–50,000,MPBiomedicals) in their drinking water *ad libitum* for 7 days, followed by 4 days of normal drinking water for recovery. Mice were euthanized on day 11 following recovery. Body weight was measured daily. After euthanasia, the intestine was removed *en bloc*, and colon length was measured. To quantify the extent of mucosal damage, a 0.5-cm segment from the distal colon was fixed in 4% paraformaldehyde, paraffin embedded, sectioned (5 um), and stained with hematoxylin and eosin staining.

### Statistical analysis

Statistical analysis was performed using the Graphpad Prism statistical software release 5.0 for Windows XP. All data were expressed as means ± SEM of data obtained from at least three independent experiments. We evaluated statistical significance with two-tailed Student's *t*-test and the 2way-ANOVA test, applying the Bonferroni post-test for the grouped analyses. Results were considered statistically significant at *p* < 0.05.

## Author contributions

MC, GS, and SD conceived and designed the experiments. GC contributed to research design and data analysis. PG and IC prepared the samples and provided experimental assistance. SD, VG, GV, ML, MM, JL, JT, AB, and MF performed the experiments. SD, AS, and AP analyzed the data. GG, PC, and GC contributed reagents, materials, and analysis tools. MC, MF, AF, and SD wrote the paper.

### Conflict of interest statement

The authors declare that the research was conducted in the absence of any commercial or financial relationships that could be construed as a potential conflict of interest.

## Supplementary material

Supplementary Figure 1 Representative amplification plot using AQP1 and AQP5 probes.

Supplementary Figure 2 IL12p70 production by HTS13286 treated DCs. Mature BMDCs were exposed to HTS13286 at the indicated times. IL-12p70 secretion was determined 24 h later by ELISA. ^*^*P* < 0.05, ^**^*p* < 0.01, ^***^*p* < 0.001.

Supplementary Figure 3 The maturational profile of mature BMDCs was impaired after HTS13286 treatment. Representative dot plot analysis of BMDCs treated with two stimulations of HTS13286 shows a mild decrease of CD11c/MHCII^+^ cells in mature BMDCs after HTS13286 treatment. x Mean values: Vehicle RPMI: 16.5; Vehicle + LPS: 23.1; HTS13286 RPMI: 15.9; HTS13286 + LPS: 18.7 (representative of 3 independent experiments).

Supplementary Figure 4 Surface markers of CD11c enriched cells from different tissues. Representative dot plot analysis of CD11c enriched cells from the spleen, peripheral lymph nodes (PLNs), mesenteric lymph nodes (MLNs) and Peyer's patches (PPs) of WT mice. **(A)** MHCII and Ly6G expression from the ungated CD11c enriched cells. **(B)** CD11c^+^ cells expression of CD11b, F4/80 and CD103.

Supplementary Figure 5 Aqp9 expression during DCs maturation. Bone marrow cells from WT mice were cultured in presence of GM-CSF and IL-4. At indicated time cells were harvested to evaluate Aqp9 expression (histogram). FACS analysis reveal MHCII, Ly6G and CD11c surface expression (dot plot). (representative of 3 independent experiments).

Supplementary Figure 6 AQP9-KO DCs expression of Aqp7 and Aqp3. The expression of *Aqp*3 and *Aqp7* was assessed in BMDCs from Aqp9-KO mice exposed to LPS for 6 or 24 h. White bar represent untreated DCs. Bars represent the mean ± SEM of 3 independent experiments. ^***^*P* < 0.001.

Supplementary Table 1 ROBs-QP treatment does not induce significant modulations in the expression of different *Aqps* in LPS-activated BMDCs. Data indicate the AVG signals of *Aqp*1-12 from 4 different donors.
