# Corrigendum: Aquaporin-9 Contributes to the Maturation Process and Inflammatory Cytokine Secretion of Murine Dendritic Cells

**DOI:** 10.3389/fimmu.2019.00216

**Published:** 2019-02-25

**Authors:** Stefania De Santis, Grazia Serino, Maria R. Fiorentino, Vanessa Galleggiante, Patrizia Gena, Giulio Verna, Marina Liso, Monica Massaro, Jinggang Lan, Jacopo Troisi, Ilaria Cataldo, Alessia Bertamino, Aldo Pinto, Pietro Campiglia, Angelo Santino, Gianluigi Giannelli, Alessio Fasano, Giuseppe Calamita, Marcello Chieppa

**Affiliations:** ^1^National Institute of Gastroenterology “S. de Bellis”, Research Hospital, Castellana Grotte, Italy; ^2^Department of Pharmacy, University of Salerno, Fisciano, Italy; ^3^Pineta Grande Hospital, Castelvolturno, Italy; ^4^Harvard Medical School Division of Pediatric Gastroenterology and Nutrition and Mucosal Immunology and Biology Research Center, Massachusetts General Hospital for Children, Boston, MA, United States; ^5^Department of Biosciences, Biotechnologies and Biopharmaceutics, University of Bari “Aldo Moro”, Bari, Italy; ^6^Department of Medicine and Surgery and Dentistry, “Scuola Medica Salernitana”, University of Salerno, Salerno, Italy; ^7^Theoreo srl-Spin-off Company of the University of Salerno, Salerno, Italy; ^8^European Biomedical Research Institute of Salerno, Salerno, Italy; ^9^Institute of Sciences of Food Production C.N.R., Unit of Lecce, Lecce, Italy

**Keywords:** AQP9, dendritic cells, inflammation, il-12, glycerol

“Patrizia Gena”, “Ilaria Cataldo” and “Giuseppe Calamita” were not included as authors in the published article. In addition, “Grazia Serino” was listed as co-last author. “Grazia Serino” is now listed as co-first author. The author list has been updated to reflect these changes. The corrected Author Contributions Statement appears below.

“MC, GS, and SD conceived and designed the experiments. GC contributed to research design and data analysis. PG and IC prepared the samples and provided experimental assistance. SD, VG, GV, ML, MM, JL, JT, AB, and MF performed the experiments. SD, AS, and AP analyzed the data. GG, PC, and GC contributed reagents, materials, and analysis tools. MC, MF, AF, and SD wrote the paper.”

Additionally, in the original article, there was an error. The authorization number “996/2015-PR” was missing for an animal experiment.

A correction has been made to **Materials and Methods, Mice:**

“WT and *Aqp9* KO (B6.129S1-Aqp9tm1Nlsn/Mmjax) murine lines were purchased from Jackson Laboratories (stocks No: 000664 and 37111, respectively). Animal experiments were carried out in accordance with Directive 2010/63/UE, enforced by Italian D.L. 26/2014, and approved by the Committee on the Ethics of Animal Experiments of the Ministero della Salute-Direzione Generale Sanità Animale (768/2015-PR 27/07/2015), the official RBM veterinarian and by the animal care and use Committee of the University of Bari (OPBA di Ateneo) and the Italian Ministry of Health (authorization n. 996/2015-PR). The Institutional Animal Care of Harvard Medical School (protocol #2013N000013) approved the protocol for the treatment of *Aqp9* KO and WT mice with DSS. Animals were sacrificed if in severe clinical conditions, in order to avoid undue suffering.”

The authors apologize for these errors and state that they do not change the scientific conclusions of the article in any way. The original article has been updated.

