# Supplementary figures and images for: Aquaporin-9 Contributes to the Maturation Process and Inflammatory Cytokine Secretion of Murine Dendritic Cells
Source: Front Immunol. 2018 Oct 16;9:2355. doi: 10.3389/fimmu.2018.02355 (PMC6198254; doi:10.3389/fimmu.2018.02355)

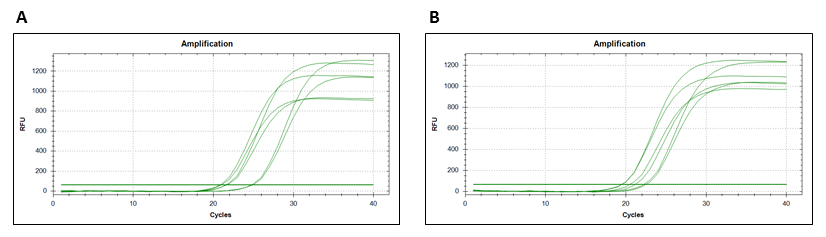

Supplement: Supplementary Figure 1 — Representative amplification plot using AQP1 and AQP5 probes. [file Image_1.TIF]

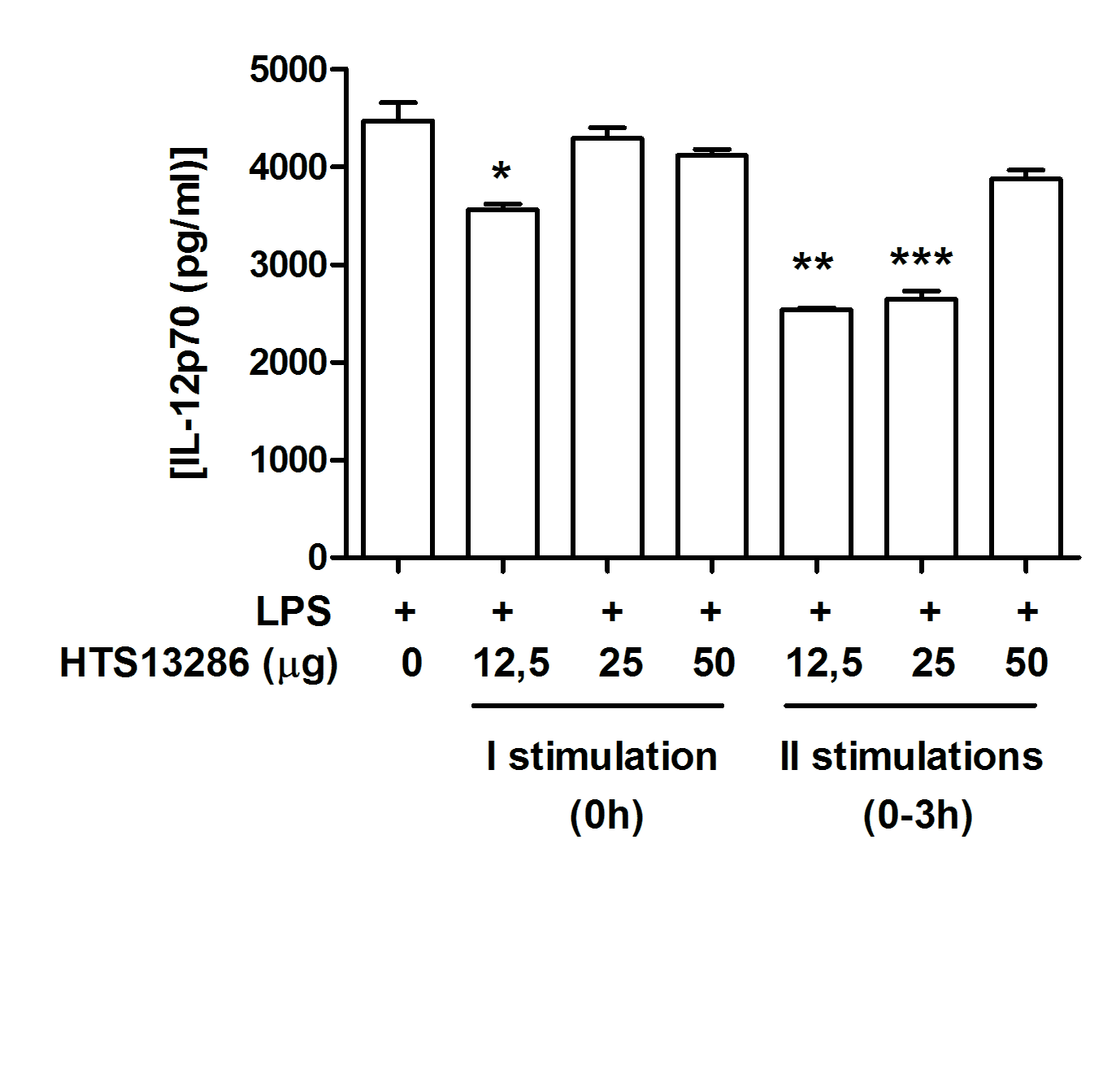

Supplement: Supplementary Figure 2 — IL12p70 production by HTS13286 treated DCs. Mature BMDCs were exposed to HTS13286 at the indicated times. IL-12p70 secretion was determined 24 h later by ELISA. *P < 0.05, **p < 0.01, ***p < 0.001. [file Image_2.TIF]

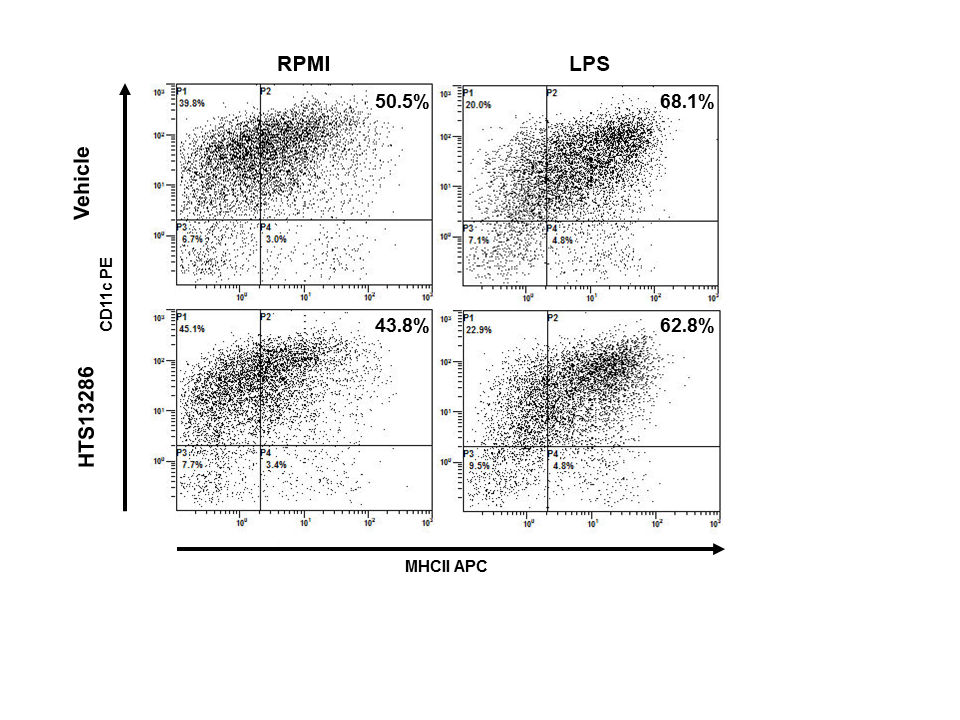

Supplement: Supplementary Figure 3 — The maturational profile of mature BMDCs was impaired after HTS13286 treatment. Representative dot plot analysis of BMDCs treated with two stimulations of HTS13286 shows a mild decrease of CD11c/MHCII+ cells in mature BMDCs after HTS13286 treatment. x Mean values: Vehicle RPMI: 16.5; Vehicle + LPS: 23.1; HTS13286 RPMI: 15.9; HTS13286 + LPS: 18.7 (representative of 3 independent experiments). [file Image_3.TIF]

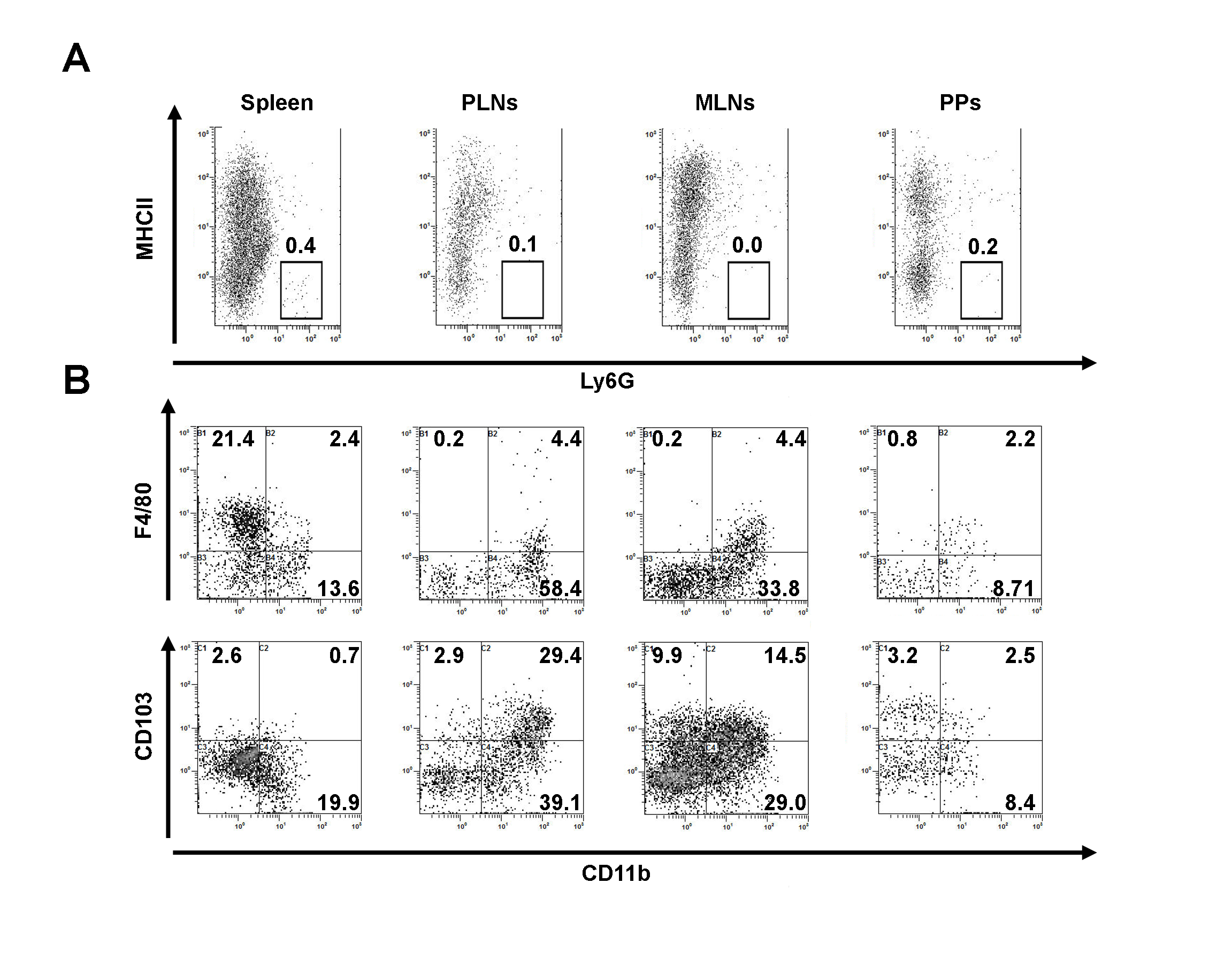

Supplement: Supplementary Figure 4 — Surface markers of CD11c enriched cells from different tissues. Representative dot plot analysis of CD11c enriched cells from the spleen, peripheral lymph nodes (PLNs), mesenteric lymph nodes (MLNs) and Peyer's patches (PPs) of WT mice. (A) MHCII and Ly6G expression from the ungated CD11c enriched cells. (B) CD11c+ cells expression of CD11b, F4/80 and CD103. [file Image_4.TIF]

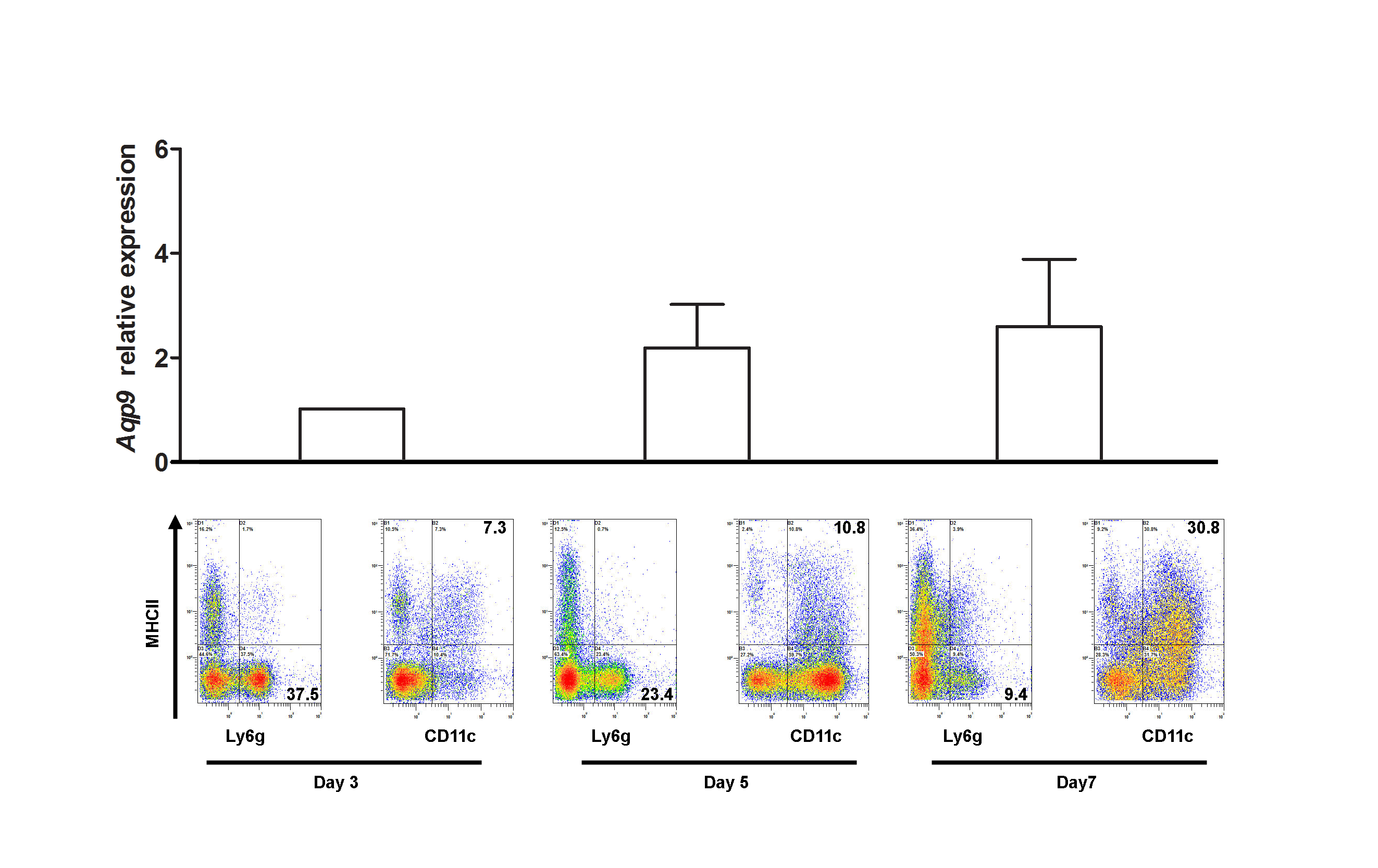

Supplement: Supplementary Figure 5 — Aqp9 expression during DCs maturation. Bone marrow cells from WT mice were cultured in presence of GM-CSF and IL-4. At indicated time cells were harvested to evaluate Aqp9 expression (histogram). FACS analysis reveal MHCII, Ly6G and CD11c surface expression (dot plot). (representative of 3 independent experiments). [file Image_5.TIF]

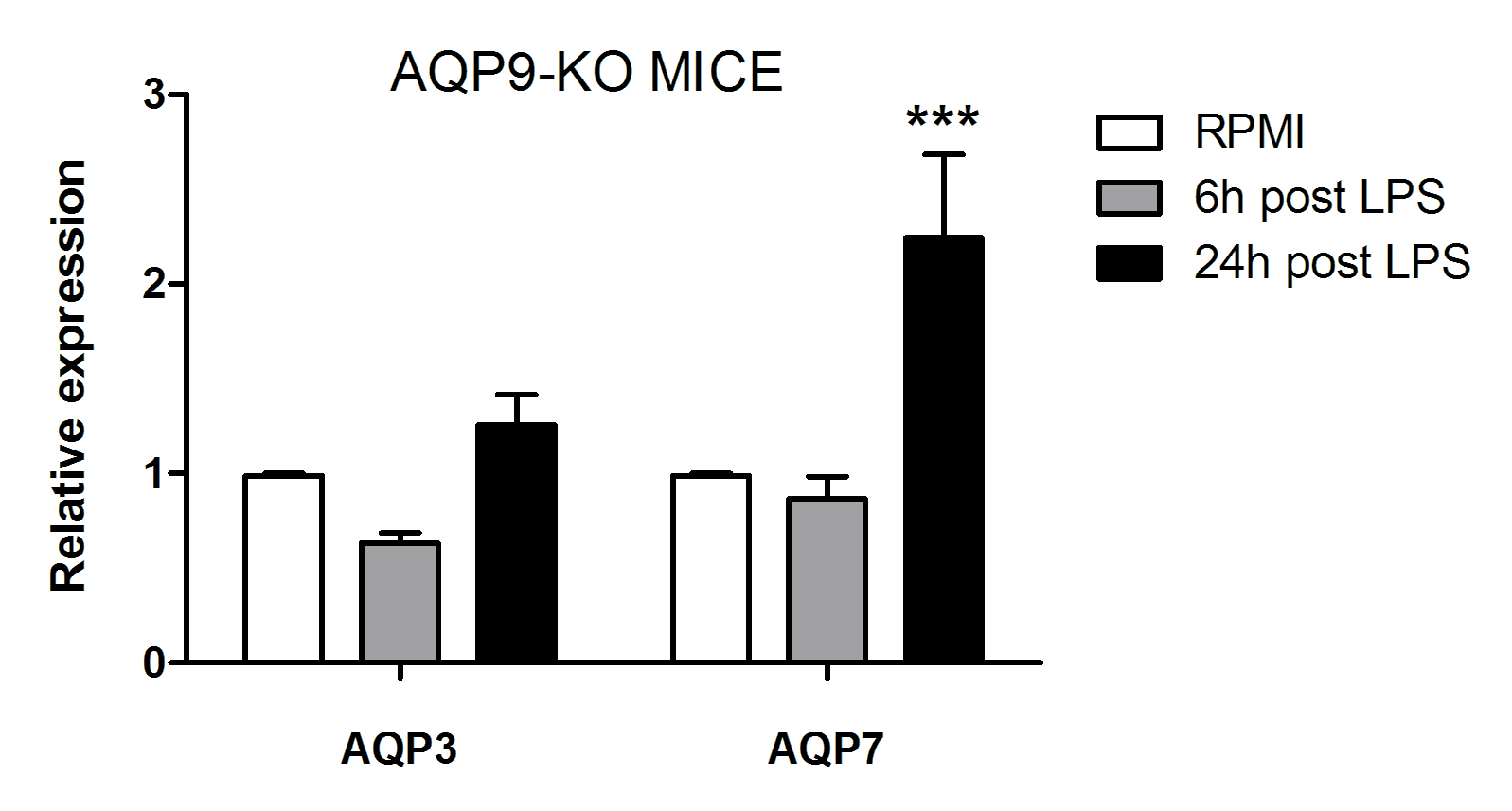

Supplement: Supplementary Figure 6 — AQP9-KO DCs expression of Aqp7 and Aqp3. The expression of Aqp3 and Aqp7 was assessed in BMDCs from Aqp9-KO mice exposed to LPS for 6 or 24 h. White bar represent untreated DCs. Bars represent the mean ± SEM of 3 independent experiments. ***P < 0.001. [file Image_6.TIF]
