# Supplementary material for: Aquaporin-9 Contributes to the Maturation Process and Inflammatory Cytokine Secretion of Murine Dendritic Cells
Source: Front Immunol. 2018 Oct 16;9:2355. doi: 10.3389/fimmu.2018.02355 (PMC6198254; doi:10.3389/fimmu.2018.02355)
Supplement: Supplementary Table 1 — ROBs-QP treatment does not induce significant modulations in the expression of different Aqps in LPS-activated BMDCs. Data indicate the AVG signals of Aqp1-12 from 4 different donors. [file Table_1.DOC]

|  | **LPS** | | | | **ROBs-QP + LPS** | | | |
| --- | --- | --- | --- | --- | --- | --- | --- | --- |
|  | **Donor 1** | **Donor 2** | **Donor 3** | **Donor 4** | **Donor 1** | **Donor 2** | **Donor 3** | **Donor 4** |
| **Aqp1** | -6,1 | 5,3 | -0,9 | -3,2 | 0,2 | 3,4 | 9,6 | -2,7 |
| **Aqp2** | 5,1 | 7,7 | 28,5 | -2,2 | 5,9 | 20,4 | 10,5 | 10,5 |
| **Aqp3** | 9,4 | 21,2 | 13,8 | 2,9 | 7,7 | 10,9 | 13,6 | 4,9 |
| **Aqp4** | -3,3 | 1,2 | -5,4 | -3,3 | 4,3 | -13,9 | -5,7 | -12,2 |
| **Aqp5** | 8,8 | 13,2 | 33,6 | -0,2 | -5,4 | 6,6 | -1,5 | 24,6 |
| **Aqp6** | 2,1 | -9,1 | -3,8 | 3,9 | -16,4 | -0,8 | -5,3 | -6,4 |
| **Aqp7** | -4,8 | -3,9 | 0,7 | -9,1 | -8,2 | 2,9 | -6,8 | 0,8 |
| **Aqp8** | 6,8 | 1,3 | 3,5 | -5,3 | 3,1 | 6,2 | 0,8 | -4,6 |
| **Aqp9** | 2180 | 2064 | 1813 | 1129 | 2842 | 3057 | 2878 | 1790 |
| **Aqp11** | 6,7 | 3,5 | 2,2 | 4,2 | 14,0 | 8,7 | 18,0 | 5,3 |
| **Aqp12** | -9,7 | -4,9 | 3,4 | -6,4 | 3,2 | -0,5 | 5,2 | -7,5 |
